# Acute Upper Respiratory Infection in a Pediatric Patient During the COVID-19 Pandemic: A Case Report

**DOI:** 10.7759/cureus.39057

**Published:** 2023-05-15

**Authors:** Joshua A Jogie

**Affiliations:** 1 Department of Medical Sciences, University of the West Indies, St. Augustine, TTO

**Keywords:** antibiotic stewardship, symptomatic management, pediatric patient, covid-19 pandemic, acute upper respiratory infection

## Abstract

Upper respiratory infections (URIs) are a frequent presentation of the COVID-19 pandemic, which has had a major detrimental impact on the pediatric population. In this case report, we detail the pandemic-related treatment of a five-year-old patient who had an acute upper respiratory illness. The case report begins with an overview of the COVID-19 pandemic, then discusses the difficulties in identifying and treating pediatric patients with respiratory illnesses in the current setting. In this report, we describe the case of a five-year-old child who originally displayed signs and symptoms of a viral URI that, with further investigation, proved unrelated to COVID-19. Treatment for the patient included symptom control, monitoring, and ultimately recovery. This study stresses the necessity for adequate diagnostic testing, individualized treatment plans, and ongoing surveillance for respiratory infections in pediatric patients during the COVID-19 pandemic.

## Introduction

The COVID-19 pandemic, caused by SARS-CoV-2, has had a profound influence, not only on the lives of millions of people worldwide but also on the treatment and management of pediatric patients [1]. Children can nevertheless have a variety of COVID-19-related symptoms and problems even, although the condition is typically less severe in them than it is in adults [2]. Additionally, the pandemic has made it more crucial to consider the similarities and differences in how COVID-19 and other pediatric respiratory infections, particularly upper respiratory infections (URIs), present [3,4].

It can be difficult for medical professionals to distinguish between COVID-19 and URIs, which are common in children and can have symptoms similar to COVID-19 [3,4]. Children's respiratory infections must be correctly diagnosed and treated to ensure adequate management and lower the risk of consequences or future transmission, especially during the current pandemic [5]. Healthcare professionals must be considerably more watchful in diagnosing and managing respiratory infections in pediatric patients and distinguishing between COVID-19 and other respiratory illnesses. This is essential as schools reopen and kids resume their regular schedules [1].

Children with COVID-19 can present clinically in a variety of ways, from mild to severe, and some cases may be asymptomatic [2]. Fever and coughing are the most frequently reported symptoms in pediatric patients. Similar symptoms are also common in other respiratory infections [3]. This overlap in clinical appearance can make diagnosis difficult and raise questions about how to treat pediatric respiratory infections during the pandemic [4].

Additionally, the COVID-19 pandemic has changed how people seek out healthcare and how they use that care, which may have an impact on how pediatric patients with respiratory infections are diagnosed and treated [5]. For instance, parents may be reluctant to take their kids to the pediatrician out of concern that they might be exposed to the virus, which could cause delays in both diagnosis and treatment [5]. Additionally, it may be difficult for healthcare providers to balance the requirement for infection control measures with the provision of prompt and appropriate care for children with respiratory infections [4].

Another crucial factor in the treatment of young children with respiratory infections during the pandemic is antibiotic stewardship, as the improper use of antibiotics might lead to the emergence of antimicrobial resistance [5]. Because fewer people are seeking medical attention for acute respiratory infections as a result of the pandemic, fewer outpatient antibiotic prescriptions have been written [5]. When treating pediatric patients with respiratory infections during the COVID-19 pandemic, healthcare professionals must nevertheless continue to follow the right antibiotic prescribing practices and take bacterial co-infections into account [5].

In this case report, we address the difficulties in diagnosing and treating a five-year-old child who had an acute URI during the COVID-19 pandemic. This instance emphasizes the significance of taking into account the similarities and differences in presentation between COVID-19 and other pediatric respiratory infections, the necessity for healthcare providers to remain vigilant in identifying and managing pediatric respiratory infections, and the function of antibiotic stewardship in ensuring appropriate treatment during the pandemic.

## Case presentation

The patient, a five-year-old female who had generally been in good health, was brought to the pediatric outpatient clinic by her parents. According to her parents, she had been experiencing symptoms for the past three days, including a continuous, high-grade fever accompanied by chills, a dry, nonproductive cough, sore throat, and nasal congestion. They clarified that her birth history was uneventful with no significant anomalies. Furthermore, her immunization records, which were up to date, reflected a routine vaccination history, indicating an otherwise standard course of health for a child her age. Before the development of these symptoms, the child had been generally healthy and had no known chronic medical issues. The family had not recently traveled, and they were not aware of any confirmed COVID-19 cases in their neighborhood or among their social contacts. 

On arrival, the patient's vital signs were as follows: temperature 38.2 °C, heart rate 110 beats per minute, respiration rate 24 breaths per minute, blood pressure 95/60 mmHg, and oxygen saturation 98% on room air. Owing to the fever and congestion, the child appeared to be adequately hydrated and in mild distress. Physical examination revealed erythematous tonsils with no exudate, tender cervical lymphadenopathy, and clear lung auscultation without wheezing, rales, or rhonchi. 

Initial laboratory tests were requested, including a complete blood count (CBC), C-reactive protein (CRP), and electrolytes, to further assess the patient's condition. No notable anomalies were found in any of the laboratory findings, which were all within normal limits. To rule out COVID-19 as the source of the child's symptoms, a nasopharyngeal swab was taken for SARS-CoV-2 polymerase chain reaction (PCR) testing. Rapid antigen testing for influenza A and B was also carried out, although the results were negative. 

The patient was administered a safe dose of acetaminophen, specifically 10 mg/kg, orally, every four to six hours as needed, for fever control - this dosage was chosen based on her weight and age. Her parents were advised to carefully monitor the dosage and duration, ensuring not to exceed five doses in 24 hours. They were also instructed to keep her well hydrated and encourage ample rest at home while awaiting the results of the SARS-CoV-2 PCR test. The family received detailed instructions on monitoring the child's symptoms and was scheduled for a follow-up visit within 48 hours, or earlier if symptoms deteriorated.

The child returned to the clinic two days later for the follow-up visit. According to the parents, the fever had subsided by this time and the child's symptoms had improved. The patient's upper respiratory illness was confirmed to be unrelated to COVID-19 by the SARS-CoV-2 PCR test result, which was negative. The doctor provided reassurance to the family, explaining that the child's illness was most likely due to a viral URI. To clarify, no bacteriological or fungal culture and sensitivity tests were performed in this case, as the clinical presentation strongly suggested a viral origin. The family was advised to continue symptomatic therapy with acetaminophen, as needed, for any residual discomfort or fever.

The family was advised to monitor the child's development and visit the clinic if the child's symptoms worsened or did not subside by the next week. If the child's condition continued to improve, there would be no need for additional monitoring. The child continued to recuperate at home, and over the following few days, their symptoms progressively became better. By the end of the next week, the child was able to resume her regular daily activities after completely recovering from the upper respiratory illness. Table 1 showcases the timeline of events in this case report.

**Table 1:** Timeline of the patient's presentation, management, and recovery. PCR, polymerase chain reaction

Day	Event
Day 1	The patient presented to the pediatric outpatient clinic
	Initial vital signs, physical examination, and laboratory tests conducted
	Nasopharyngeal swab for SARS-CoV-2 PCR and rapid antigen testing for influenza A and B performed
	Prescribed acetaminophen and rest and encouraged to remain well hydrated
Day 3	The patient's follow-up appointment
	Fever resolved and symptoms improved
	SARS-CoV-2 PCR test result returned negative
	Advised to continue symptomatic treatment and follow up if symptoms worsened or did not resolve within the next week
Day 10	The patient fully recovered

## Discussion

The COVID-19 pandemic has made it more difficult to diagnose and treat pediatric patients' respiratory infections because of similar symptomatology [6]. However, symptomatic treatment approaches are often used to treat other common viral respiratory tract infections, such as influenza, respiratory syncytial virus (RSV), rhinovirus, and adenovirus. These could include rest to encourage recovery, increased fluid intake to prevent dehydration, and the control of fever with drugs such as acetaminophen or ibuprofen [7]. In some cases, doctors may recommend antiviral drugs for influenza, such as oseltamivir. In situations of severe bronchiolitis, typically brought on by RSV, nebulization and inhalation treatment may be employed. Antibiotics should only be administered if a subsequent bacterial infection is suspected because they are ineffective against viruses [8].

In this case, the patient displayed symptoms of an upper respiratory viral illness, which can indicate COVID-19 or another viral infection [9]. The ability to distinguish COVID-19 from other respiratory diseases through the use of PCR testing allows for effective management [10].

The control of symptoms and supportive care are the main components of treatment for viral URIs in children. To aid with recuperation, it is essential to make sure that children obtain adequate nutrition, hydration, and rest [11]. Additionally, medical professionals might suggest drugs to treat symptoms, including fever, cough, or nasal congestion. Antibiotics should only be used to treat bacterial infections; they are not recommended for viral infections [12].

There are disputes surrounding the use of over-the-counter (OTC) cough and cold drugs in children under the age of six. Some studies indicate that these OTC medications may have poor efficacy and pose potential safety risks [13]. Due to potential side effects, the American Academy of Pediatrics (AAP) advises against giving OTC cough and cold drugs to children under the age of four [7]. Instead, symptomatic relief for younger children can be obtained via supportive measures, including saline nasal drops, nasal suctioning, and humidity control [14].

It is crucial to take into account the potential repercussions of SARS-CoV-2 infection in children who present with signs of an upper respiratory illness in the context of the COVID-19 pandemic. Children can still spread the COVID-19 virus to populations who are at risk, although their symptoms are often less severe than those of adults [15]. Therefore, it is essential to abide by public health recommendations, which include getting tested for COVID-19, maintaining good hygiene, and following quarantine or isolation protocols as needed [16].

In this instance, the patient's symptoms and presentation were suggestive of an acute URI. However, it was essential to take into account the possibility of SARS-CoV-2 infection given the ongoing COVID-19 pandemic. A COVID-19 test was performed on the patient, and the results were negative. As a result, the medical professional was able to focus on providing supportive care and managing symptoms without the risk of COVID-19 complications or transmission.

The patient's treatment plan included adequate rest, nutrition, and hydration. The medical staff advised using OTC acetaminophen to treat the patient's fever and pain. To relieve nasal congestion, the parents were also advised on suctioning and the use of saline nasal drops. The patient was kept under close observation for any signs of deteriorating symptoms or potential bacterial complications, such as persistent fever, respiratory difficulties, or symptoms of ear infection [17].

The author acknowledges the need to contribute additional original research to the field. Additionally, the author values the reminder that other factors, besides COVID-19, should be taken into account when determining the cause of respiratory infections. In the future, case reports should concentrate on providing more thorough patient histories and treatment regimens, including particular prescription dosages and durations and extensive immunization data. In addition, despite the disruption brought on by the current epidemic, it is imperative to stress the significance of a complete evaluation for each patient. Although the case described here is neither exceptional nor particularly novel, it serves to highlight once again how crucial routine diagnostics are in differentiating between COVID-19 and other prevalent viral respiratory diseases. This makes it possible to manage patients properly and avoid unnecessary COVID-19-related transmission or complications.

## Conclusions

The challenges involved in managing acute URIs in children during the continuing COVID-19 pandemic are highlighted by this case report. The patient's symptoms were in keeping with an upper respiratory viral infection; hence, the possibility of SARS-CoV-2 infection had to be taken into account. The healthcare team was able to focus on symptomatic therapy and supportive care because the COVID-19 test was negative, eliminating any concerns about COVID-19 implications or transmission.

The recommended course of treatment included providing appropriate hydration, rest, and nutrition as well as acetaminophen for pain relief and fever control that was available OTC. Nasal suctioning and saline nasal drops were used to treat congestion. The patient was kept under strict observation for any indications of deteriorating symptoms or possible bacterial complications.

This case report serves as a reminder of the value of a comprehensive assessment and attention to public health recommendations while treating URIs in children during the COVID-19 epidemic. The management of symptoms and supportive care should be the main priorities, and drugs should be used carefully to minimize any potential side effects. To provide the best outcomes for their pediatric patients, healthcare professionals must also be cautious in monitoring for complications and taking SARS-CoV-2 infection into account.
